# Various Techniques to Increase Keratinized Tissue for Implant Supported Overdentures: Retrospective Case Series

**DOI:** 10.1155/2015/104903

**Published:** 2015-06-01

**Authors:** Ahmed Elkhaweldi, Carmen Rincon Soler, Rodrigo Cayarga, Takanori Suzuki, Zev Kaufman

**Affiliations:** ^1^Department of Periodontology and Implant Dentistry, New York University College of Dentistry, 345 East 24th Street, New York City, NY 10010, USA; ^2^Universidad Francisco Marroquín, 01010 Guatemala City, Guatemala

## Abstract

*Purpose*. The purpose of this retrospective case series is to describe and compare different surgical techniques that can be utilized to augment the keratinized soft tissue around implant-supported overdentures. *Materials and Methods*. The data set was extracted as deidentified information from the routine treatment of patients at the Ashman Department of Periodontology and Implant Dentistry at New York University College of Dentistry. Eight edentulous patients were selected to be included in this study. Patients were treated for lack of keratinized tissue prior to implant placement, during the second stage surgery, and after delivery of the final prosthesis. *Results*. All 8 patients in this study were wearing a complete maxillary and/or mandibular denture for at least a year before the time of the surgery. One of the following surgical techniques was utilized to increase the amount of keratinized tissue: apically positioned flap (APF), pedicle graft (PG), connective tissue graft (CTG), or free gingival graft (FGG). *Conclusions*. The amount of keratinized tissue should be taken into consideration when planning for implant-supported overdentures. The apical repositioning flap is an effective approach to increase the width of keratinized tissue prior to the implant placement.

## 1. Introduction

Dental implant-supported overdentures have been documented to be a predictable and successful option to treat edentulous patients [1, 2]. Currently, with the evolution of implant surfaces, osseointegration of implants is less of a challenge [3]. However, the stability and health of the peri-implant soft tissue is necessary for the success and the long-term maintenance of dental implants [4]. Two millimeters wide band of keratinized tissue has been considered clinically desirable to provide a soft tissue seal around natural teeth [5]. However, controversy still remains over the necessity for a band of keratinized tissue around dental implants [6–9]. The role of dental plaque in the etiology of peri-implant diseases is well documented in the literature [10, 11]. The absence of periodontal ligament, supracrestal fibers attachment around dental implants may make peri-implant tissue more susceptible to an inflammatory process caused by plaque accumulation [12].

Several studies have reported increased gingival and plaque index scores, mucosal recession, and marginal bone resorption in areas around implants with less than 2 mm of keratinized tissue [4, 8, 13–16]. Conversely, some authors have claimed that, with adequate plaque control, peri-implant tissues can be maintained in a healthy state with a minimum amount of keratinized tissue [6–9].

However, patient discomfort has been reported to be associated with insufficient keratinized tissue in implant-supported overdentures [17]. In many cases, performing oral hygiene was reported to be painful as a result to the absence of the keratinized tissue surrounding the implant. Moreover, discomfort has been related to mechanical irritation due to the mobility of the nonkeratinized tissue under function [17, 18].

In 1999, Kaptein et al. investigated the peri-implant tissue health of loaded implants. There was a significantly higher gingival index and probing depth in overdenture versus fixed prosthesis cases [18]. It has been reported that implants supporting overdentures had more risk for bone loss, based on poorer peri-implant tissue health [18]. Adibrad et al. investigated the association between the width of the keratinized tissue and the health status of the soft tissue around implants supporting overdentures. They concluded that the absence of adequate keratinized tissue was associated with a higher plaque accumulation, gingival inflammation, bleeding on probing, and mucosal recession [17].

To date, there are a limited number of studies that discuss peri-implant tissue health and the presence of keratinized tissue around implants supporting overdentures [17–19]. These studies conclude that the presence of keratinized tissue around implant-supported overdentures is a factor effecting bone maintenance and soft tissue health around those implants [17, 18].

Various surgical procedures have been developed to preserve and/or reconstruct keratinized tissue around dental implants [20–24]. These techniques, including apically positioned flaps, pedicle grafts, free gingival grafts, and connective tissue grafts, can be performed prior to implant placement, during the second stage surgery or after delivery of the final prosthesis. Allogenic and xenogenic soft tissue grafts have also been used as other options for increasing peri-implant keratinized tissue [24–27].

The purpose of this retrospective case series was to describe and compare different surgical techniques that can be utilized to augment the keratinized soft tissue around dental implant-supported overdentures.

## 2. Materials and Methods

Clinical data in this study was obtained from implant database (ID). This data set was extracted as deidentified information from the routine treatment of patients at the Ashman Department of Periodontology and Implant Dentistry at New York University College of Dentistry. The ID was certified by the Office of Quality Assurance at NYUCD. This study is in compliance with the Health Insurance Portability and Accountability Act (HIPAA) requirements.

### 2.1. Study Subjects

Eight edentulous cases were selected from the ID to be included in this retrospective study. Patients were treated for lack of keratinized tissue prior to implant placement, during the second stage surgery or after delivery of the prosthesis. The population consisted of 2 females and 6 males, with a mean age of 65 years (range: 54 to 83). In 7 out of 8, the augmentation procedure was performed in the mandible.

### 2.2. Inclusion Criteria

1. Patients who underwent implant surgery and were restored with a maxillary and/or mandibular implant-supported overdenture.
2. Patients wearing a maxillary and/or mandibular implant-supported overdenture.
3. Clinical symptoms of discomfort or difficulty to perform oral hygiene due to insufficient keratinized tissue around implant-supported overdentures. Insufficient was defined as <2 mm of keratinized gingiva.
4. Patients who underwent a surgical procedure to increase keratinized tissue around implant-supported overdentures.

### 2.3. Exclusion Criteria

1. Presence of systemic diseases that influence bone or soft tissue metabolism.
2. Smoking habit of more than a pack a day, and unwillingness to stop.
3. Radiotherapy to head/neck region in the past 12 months prior to surgery.
4. Chemotherapy in the past 12 months prior to surgery.
5. Unwillingness to commit to a long-term maintenance program after treatment.

### 2.4. Description of the Protocol

1. Preoperative measurement of the width of keratinized tissue, in the area of the planned implants, or around already placed implants, measured in millimeters using a periodontal probe from the free soft tissue margin to the mucogingival junction. All the measurements were performed by the same investigator.
2. Antibiotic premedication: 2 g Amoxicillin 1 hour prior to surgery or 600 mg Clindamycin in case of penicillin allergy.
3. Infiltrative local anesthesia using Lidocaine HCl 2% containing epinephrine 1 : 100,000 or Carbocaine 3% without epinephrine in cases where a vasoconstrictor was contraindicated.
4. One of the following techniques was utilized to increase the amount of keratinized tissue: apically positioned flap, pedicle graft, connective tissue graft, or free gingival graft. The technique was selected depending on the time the surgery was performed and operator preferences (Table 1).
5. Postsurgically, the patient was instructed to not wear their prosthesis for 3 weeks.
6. Postoperative antibiotics (Amoxicillin 500 mg tid or Clindamycin 150 mg qid) and analgesics (Ibuprophen 600 mg q 4–6 hrs) were prescribed for a week.
7. Postoperative care instructions were given, including use of Clorhexidine gluconate 0.12% rinses 3 times a day and soft diet, for two weeks.
8. Postoperative measurement of the width of keratinized tissue taken 1 month and 3 months after the surgery, using a periodontal probe and measured from the free gingival margin to the mucogingival junction in the area where the preoperative measurement was taken and where the surgical technique was performed. Photos of the surgical procedure were used to duplicate the area of measurement.

## 3. Results and Discussion

Over time, clinicians have used different surgical techniques to increase the width of keratinized tissue around natural teeth. These techniques have also been applied around implant-supported restorations. Each of these techniques has advantages and limitations. Understanding these techniques would help the clinician to decide which one to use in specific circumstances. In this study, fifteen sites in eight patients were treated to increase the amount of keratinized tissue. All 8 patients in this study were wearing a complete maxillary and/or mandibular denture for at least a year before the time of the surgery. One of the following surgical techniques was utilized to increase the amount of keratinized tissue: apically positioned flap (APF), pedicle graft (PG), connective tissue graft (CTG), or free gingival graft (FGG). In seven out of the eight cases, the surgery was performed in the mandible. The augmentation procedure was performed on three cases with the implants already restored with the final prosthesis. Four cases had the procedure done as part of the second stage implant surgery. However, in one case the augmentation utilized before the implants were placed.

**Figure 1:**
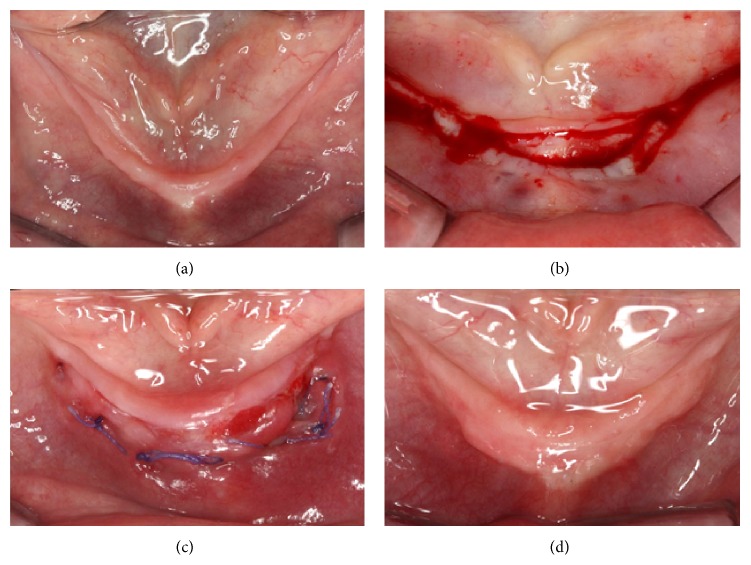
(a) Initial clinical appearance of the mandibular ridge with 3-4 mm of keratinized tissue. (b) Crestal horizontal beveled incision made. (c) The flap sutured apically with Vicryl 4.0 to the periosteum. (d) Final result 3 months after the surgery showed 5 mm increase in the width of keratinized tissue.

When planning for implant-supported overdentures, a preoperative assessment of the amount of keratinized tissue is an important step. When necessary, augmentation of keratinized mucosa should be done prior to implant placement. In case 1, an apically positioned flap was performed one month before the stage 1 surgery to allow adequate soft tissue closure (Figure 1). The initial measurement of the band of keratinized tissue in sites #22 and #27 was 3 and 4 mm, respectively. A single horizontal beveled incision was made into the attached gingiva (Figure 1(b)). The mesiodistal extension of the incision was made from #21 to #28, making it possible to elevate a partial thickness flap which was apically repositioned by suturing the flap to the periosteum with Vicryl 4.0 (Polyglactin 910) (Figure 1(c)). As a result of this procedure, a 5 mm increase in the width of keratinized tissue was obtained at both sites (Figure 1(d)). When a surgery to increase the width of keratinized tissue is performed during implant placement, the incision should be designed to maintain the amount of keratinized tissue. This incision design will allow the implant to be surrounded by at least 2 mm of keratinized tissue all around.

**Figure 2:**
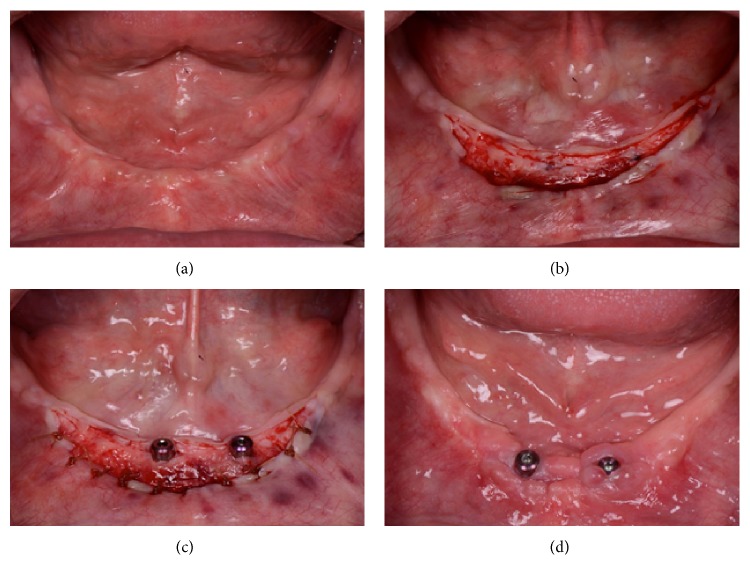
(a) Presurgical appearance of mandibular ridge with 0-1 mm of keratinized tissue. (b) Partial thickness flap reflection. (c) Apical suturing of the flap to the periosteum using Chromic Gut 4.0. (d) Final result after the surgery showed a 2-3 mm increase in keratinized tissue.

**Figure 3:**
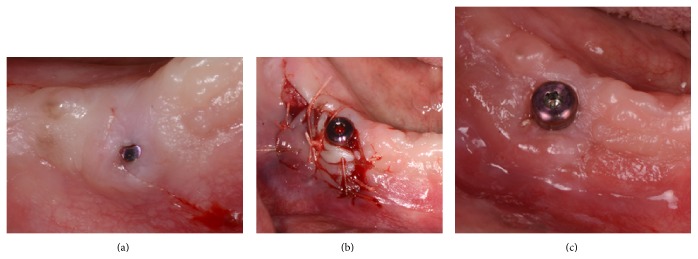
(a) Presurgical appearance of an implant supporting overdenture, 1 mm keratinized tissue. (b) Pedicle graft elevated and suture buccally. (c) Final result with 3 mm gain of keratinized tissue.

A second stage surgery is a good opportunity to increase the width of keratinized tissue (Figures 2(a), 2(b), 2(c), and 2(d)). This approach was utilized in cases 2, 3, and 4. In three patients, an apically repositioned flap was used as described in case 1, which resulted in a mean increase in the width of keratinized tissue of 3.1 mm. Case 5 was also treated as part of the second stage surgery utilizing pedicle flap with a mean increase of 2.8 mm. The pedicle flap technique is an approach similar to an apically repositioned flap and should be used when there is adequate keratinized tissue adjacent to the implant. A beveled horizontal incision of approximately 6 mm was made distal to the implant, with a small vertical incision at the distal end part of the first incision. A partial thickness flap was then elevated and the pedicle flap sutured apically (Figures 3(a), 3(b), and 3(c)).

**Figure 4:**
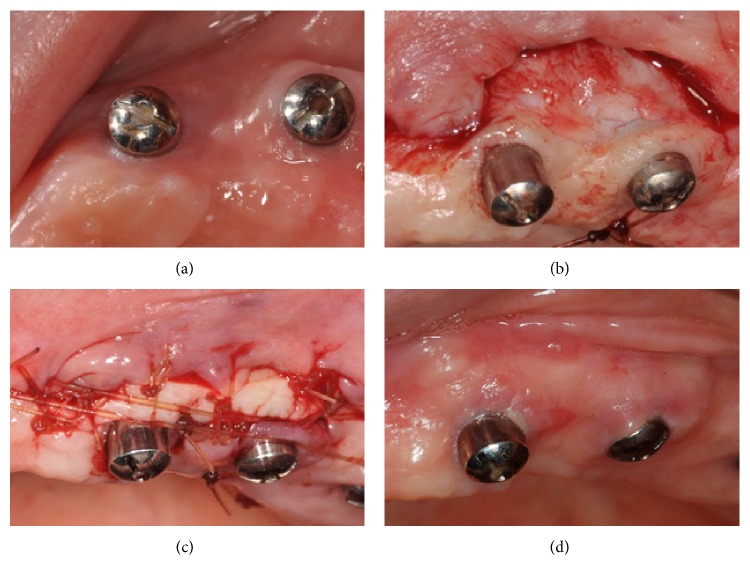
(a) Presurgical appearance of two implants with 1 mm of buccal keratinized tissue. (b) Partial thickness flap on the recipient bed prepared for the FGG. (c) Interrupted and horizontal mattress sutures to stabilize the FGG obtained from the palate. (d) Three-month follow-up showing an increase of 2-3 mm of keratinized tissue.

In some cases, a lack of keratinized tissue is evident after the insertion of the final prosthesis, causing discomfort and restricting oral hygiene performance. Moreover, since implant-supported overdentures are a removable prosthesis, patients often experience pain when taking the overdenture on and off. In this retrospective case series, three patients had surgery to increase the amount of keratinized tissue around 6 implants supporting overdentures, either by utilizing free gingival grafts or connective tissue grafts. The selection was based on the anatomy of the palate. Preference was giving to connective tissue graft when the patient had high vault palate, which allows harvesting a good amount tissue and reduces the risk of endangering the greater palatine artery. In case 6, an autogenous free gingival graft was harvested from the palatal premolar area, around #12, 13, and then sutured to the periosteal recipient bed of #11 and #13 (Figures 4(a), 4(b), and 4(c)). After healing and maturation of the soft tissue an increase of 3 and 2 mm was obtained, respectively, (Figure 4(d)). Cases 7 and 8 were treated with connective tissue grafts harvested from the premolar area of the palate. At the same time, the recipient site was prepared; a vertical incision mesial to the implant was made and a partial thickness flap was then elevated, creating a tunnel where the connective tissue graft was inserted and sutured. One of them (case 7) resulted in no increase of keratinized tissue as a result of significant decrease of the vestibular depth following the excessive amount of alveolar bone resorption. In case 8, the healing was accompanied with nonkeratinized soft tissue growth over the implant which made it very difficult both to perform oral hygiene and to insert the overdenture. A customized healing abutment was designed to control the excessive growth, and two more implants were placed, converting the overdenture prosthesis.

Each of the soft tissue augmentation techniques has advantages and limitations. The apically repositioned flap is a relatively simple procedure that provides a good esthetic outcome, as the newly formed tissue is indistinguishable from the surrounding mucosa. Moreover, shorter operative time and low morbidity is involved [20]. The main limitation of this technique is the need for at least 0.5 mm millimeters of keratinized tissue preoperatively. In cases where less than 0.5 mm of keratinized tissue is present preoperatively, autogenous free gingival grafts present an effective option. Free gingival grafts have been proven to be successful and predictable. However, these also present disadvantages. They involve two surgical sites with the consequent morbidity in both areas. Moreover, discrepancies in color and texture with the surrounding mucosa oftentimes result in a compromised esthetic outcome [24]. When using these techniques, some percentage of shrinkage should be expected. After one year, it has been reported that in the case of a free gingival graft, shrinkage of 38 to 45% occurs in relation to the thickness of the graft [28]. This shrinkage is even greater in cases where acellular dermal allografts are used [24]. Connective tissue graft was utilized in two cases. Although the augmentation was not successful in one case, this technique can still be an option to augment the keratinized tissue around implants restorations. There was average of 1.5 mm increase in the width of the keratinized tissue. Zucchelli et al. reported a similar result for CTG around single implant restoration. However, the author believes that the stability of the graft is very important for this technique to be successful. Pedicle Graft was utilized in one case as part of second stage surgery. This technique was less invasive and resulted in up to 3 mm increase in the keratinized tissue. This technique can be very useful in unilateral single implant cases where only small areas of narrow keratinized tissue need to be augmented [29].

## 4. Conclusions

The amount of keratinized tissue should be taken into consideration when planning for implant-supported overdentures. When the initial amount is considered insufficient, surgical augmentation procedures should be performed. An apical repositioning flap is an effective approach to increase the width of keratinized tissue prior to the implant placement if 0.5 mm of keratinized tissue was preoperatively available. During the second stage surgery, lingualized incision designs and pedicle grafts are a less invasive alternative to increase a limited zone of keratinized mucosa. Although free gingival graft or connective tissue graft could also be utilized but around implants, they can impose some challenges to the clinician during the surgery or throughout the healing. When patients experience discomfort after insertion of the final prosthesis due to a lack of keratinized mucosa, free gingival or connective tissue grafts are a feasible alternative. In some cases, a change of design of the prosthesis could be performed, placing more implants and converting from overdenture to a fixed restoration.

## Figures and Tables

**Table 1:** Gain in keratinized tissue three months after surgical procedure.

Case	Gender	Age	Site	KT initial (mm)	KT final (mm)	Increase KT (mm)	Time of surgery	Technique
1	Male	65	#22	3	8	5	Prior to implant placement	APF
			#27	4	9	5		
2	Male	72	#22	0-1	4	3-4	2nd stage	APF
			#27	1-2	5	3-4		
3	Female	54	#22	1	4	3	2nd stage	APF
4	Male	59	#22	1	4	3	2nd stage	APF
			#27	0-1	3	2-3		
5	Male	61	#22	1	3	2	2nd stage	PG
			#27	1	4	3		
6	Male	60	#11	1	4	3	After	FGG
			#13	1	3	2		
7	Female	65	#22	1	2-3	1-2	After	CTG
			#27	1	2-3	1-2		
8	Male	83	#22	0-1	0-1	0	After	CTG
			#27	0-1	0-1	0		
